# Comparison of 1-Palmitoyl-2-Linoleoyl-3-Acetyl-Rac-Glycerol-Loaded Self-Emulsifying Granule and Solid Self-Nanoemulsifying Drug Delivery System: Powder Property, Dissolution and Oral Bioavailability

**DOI:** 10.3390/pharmaceutics11080415

**Published:** 2019-08-16

**Authors:** Dong Shik Kim, Jung Suk Kim, Soo-Jeong Lim, Jong Oh Kim, Chul Soon Yong, Han-Gon Choi, Sung Giu Jin

**Affiliations:** 1 College of Pharmacy, Hanyang University, 55 Hanyangdaehak-ro, Sangnok-gu, Ansan 15588, Korea; 2 Department of Bioscience and Biotechnology, Sejong University, Gunja-Dong, Seoul 143-747, Korea; 3 College of Pharmacy, Yeungnam University, 214-1, Dae-Dong, Gyongsan 38541, Korea; 4 Department of Pharmaceutical Engineering, Dankook University, 119 Dandae-ro, Dongnam-gu, Cheonan 31116, Korea

**Keywords:** oily liquid drug, 1-palmitoyl-2-linoleoyl-3-acetyl-rac-glycerol, self-emulsifying granule system, fluid bed granulation, flow ability, oral bioavailability

## Abstract

The main objective of this study was to compare the powder property, dissolution and bioavailability of 1-palmitoyl-2-linoleoyl-3-acetyl-rac-glycerol (PLAG)-loaded self-emulsifying granule system (SEGS) and solid self-nanoemulsifying drug delivery system (SNEDDS). Various SEGS formulations were prepared, and the effect of surfactant and binder on the drug solubility in them, leading to selecting sodium lauryl sulphate (SLS) and hydroxyl propyl methyl cellulose (HPMC). The SEGS and SNEDDS were prepared with PLAG/SLS/HPMC/calcium silicate/microcrystalline cellulose at the weight ratio of 1:0.25:0.1:0.5:3 employing the fluid bed granulation and spray-drying technique, respectively. Their powder properties were compared in terms of flow ability, emulsion droplet size, scanning electron microscopy, and powder X-ray diffraction. Furthermore, the solubility, dissolution, and oral bioavailability in rats of the SEGS were assessed in comparison with the SNEDDS. The SEGS and SNEDDS enhanced the solubility of the drug approximately 36- and 32-fold as compared with the drug alone; but they had no differences. The crystalline drug may exist in both the calcium silicate and microcrystalline cellulose (MCC) in the SEGS, but only in the calcium silicate in the SNEDDS. The SEGS had considerably improved the flow ability (Hausner ratio, 1.23 vs. 1.07; Carr index, 19.8 vs. 43.5%) and drug dissolution as compared with the SNEDDS. The SEGS and SNEDDS with double peak profiles, unlike the single peak of drug alone, showed a significantly higher plasma concentration and area under the curve (AUC), as compared with drug alone. Although they were not significantly different, the SEGS gave higher AUC than did the SNEDDS, suggesting its enhanced oral bioavailability of PLAG. Thus, the SEGS could be used as a powerful oral dosage form to improve the flow ability and oral bioavailability of PLAG, an oily drug.

## 1. Introduction

**Figure 1:**
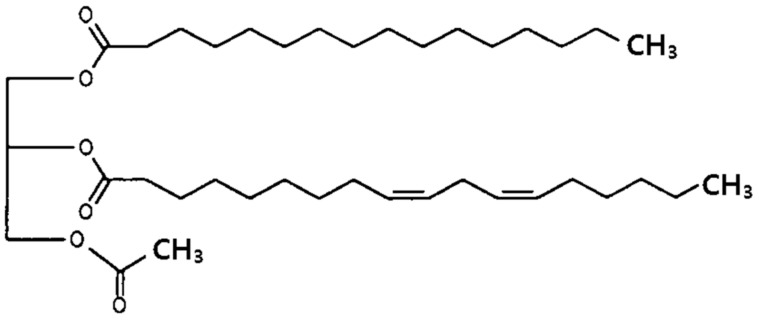
Structure of 1-palmitoyl-2-linoleoyl-3-acetyl-rac-glycerol (PLAG).

The oily drug, 1-palmitoyl-2-linoleoyl-3-acetyl-rac-glycerol (PLAG, Figure 1), was originally isolated from seed oils, bovine udder, and deer horns. PLAG is chemically synthesized by reacting palmitic acid, linoleic acid, and glycerol [1]. PLAG has been shown to modulate eosinophil chemotaxis in epithelial cells and to effectively suppress neutrophilic inflammation [2]. Moreover, the United States Food and Drug Administration (FDA) has approved phase II clinical trials of PLAG for the management of severe chemotherapy-induced neutropenia in patients with advanced breast cancer who are receiving intermediate febrile neutropenia-risk chemotherapy [3]. PLAG has a poor aqueous solubility, and thus, its dissolution rate limits absorption [4].

Various solubility and bioavailability enhancement approaches, such as solid dispersion [5], micronization [6], permeation enhancers [7], use of surface-active agents [8], pH modification [9] and self-emulsifying drug delivery systems [10], have been applied to poorly water-soluble drugs. Hence, due consideration has been given to self-emulsifying drug delivery systems that are easily manufactured for oily drugs [11]. The self-emulsifying drug delivery systems, drug-loaded blends of oils, surfactants, and co-surfactants, can be used to increase dissolution and absorption due to the spontaneous formation of a submicron droplet size. Generally, conventional self-emulsifying drug delivery systems are filled into soft gelatin capsules (liquid form) and are associated with problems related to production costs, stability, storage, and reproducibility [12]. These difficulties can be avoided by preparing solid self-emulsifying drug delivery systems that focus on the incorporation of liquid and semi-solid to solid forms, employing various solidification processes, such as adsorption to solid carriers [13], spray-drying [14], and nanoparticle technology [15]. Low flow ability in the final solid self-emulsifying drug delivery system can regularly be obtained following conversion of the drug to powder form [16]. However, the self-emulsifying granule system (SEGS), the granule form of solid self-emulsifying drug delivery systems, has increased bulk density and flow ability for preparing oral dosage forms [17]. The fluid bed granulation process is a versatile approach for obtaining solid granule forms [18].

Here, we aimed to compare the powder property, dissolution and bioavailability of two different systems, SEGS and SNEDDS, for improving the flow ability and oral bioavailability of PLAG, a poorly water-soluble oily drug. The SEGS were prepared using fluid bed granulation. PLAG-loaded SNEDDS was also prepared with the same SEGS composition using a spray-drying method for comparison. The solubility of PLAG was determined in SEGS prepared by varying the concentrations of the solid surfactant (sodium lauryl sulphate (SLS) and poloxamer), binder, calcium silicate, and microcrystalline cellulose (MCC). The SEGS and SNEDDS were evaluated by in vitro dissolution and solid-state characterization by flow ability analysis (repose angle, Carr index and Hausner ratio), powder X-ray diffraction (PXRD), and scanning electron microscopy (SEM). Furthermore, the oral bioavailability studies were conducted in rats to determine the oral bioavailability of PLAG from the optimized SEGS, in comparison with the drug alone and the SNEDDS.

## 2. Materials and Methods

### 2.1. Materials

PLAG was kindly provided by Enzychem Co., (Seoul, Korea). Polyvinylpyrrolidone (PVP^®^, K30), Poloxamer^®^ 407 (poloxamer), and Microcrystalline cellulose (MCC, Avicel^®^ PH-101) were provided by BASF (Ludwigshafen, Germany). Hydroxypropyl cellulose (HPC-L^®^), hydroxypropyl methylcellulose 15000 (HPMC), and sodium carboxymethyl cellulose (sodium CMC) were provided by Shin-Etsu Co., (Tokyo, Japan). Polyvinyl alcohol (PVA) was obtained from Sigma-Aldrich Co., (St. Louis, MO, USA). Carbopol^®^ was obtained from Lubrizol (Cleveland, OH, USA). Sodium lauryl sulphate (SLS) and calcium silicate were obtained from Hanmi Pharmaceutical Co., (Suwon, Korea). All other chemicals and solvents were of reagent grade and used without further purification.

### 2.2. Animals

Healthy male Sprague Dawley rats, weighing 250–300 g, were obtained from Nara Biotech (Seoul, Korea), and acclimatized and maintained at 25 ± 2 °C/50–60% relative humidity prior to experimentation. Prior to the study, the rats were kept on overnight fasting with free access to drinking water. The protocol for animal experimentation was implemented in accordance with NIH Policy and the Animal Welfare Act under the approval of the Institutional Animal Care and Use Committee (IACUC) at Hanyang University (Permission No.: HY-IACUC-2018-0007, 13 February 2018).

### 2.3. Solubility Tests

Solubility tests of PLAG were performed according to a previously published method [19]. An excess of PLAG was added to 10 mL distilled water containing different amounts of SLS or poloxamer (0.05–0.5 g) and 0.05 g various binder polymers on 0.125 g SLS or poloxamer. These solutions were placed in a temperature-controlled shaking water bath at 25 °C for 7 days, and the resulting suspensions were centrifuged at 13,000× *g* for 10 min. The quantitative analysis of PLAG was performed using an HPLC system (1220 module; Agilent, Santa Clara, CA, USA). Chromatographic separations were carried out using an Agilent Zorbax Eclipse XDB C-18 column (250 mm, 4.6 mm ID, 5 μm). The mobile phase was a mixture of acetonitrile-isopropanol (55:45, volume ratio). The effluent was monitored at a UV wavelength of 210 nm and a flow rate of 1.0 mL/min. The column temperature was maintained at 30 °C [20]. Moreover, a solubility test was also carried out for all SEGS and SNEDDS formulations.

### 2.4. Preparation of SEGS and SNEDDS

#### 2.4.1. Preparation of SEGS by Fluid Bed Granulation 

The SLS (surfactant) and HPMC (binder) were selected through solubility testing. The spray solution was prepared as 1 g PLAG and different amounts of SLS and HPMC in 500 mL ethanol-water (1:9, volume ratio) with thorough stirring for 1 h. Prior to granulation, the fluid bed excipient (various ratios of calcium silicate and MCC) was first introduced inside the unit and fluidized by ascendant airflow for 30 min to equilibrate the temperature, followed by spraying (3 mL/min) using a top-spray mode. In preliminary experiments to optimize the conditions for the granulation process, SEGS were spray-dried using a fluid bed granulator (Mini-Glatt, Glatt GmbH, Germany) with the following granulation process parameters: inlet temperature = 65 ± 5 °C; static inlet pressure = 0.5 bar; and product temperature = 35 ± 5 °C.

#### 2.4.2. Preparation of SNEDDS by Spray-Drying Method

The spray solution was prepared as 1 g PLAG and optimized amounts of SLS and HPMC in 500 mL ethanol-water (1:9, volume ratio). In a similar experiment, calcium silicate (0.5 g) and MCC (3 g) were suspended in the resulting solutions. In preliminary experiments to optimize the conditions for the spray-drying process, SNEDDS was prepared using a Büchi B-290 nozzle-type mini spray-dryer (Flawil, Switzerland) with the following process parameters: flow rate = 3 mL/min; inlet temperature = 65 ± 3 °C; outlet temperature = 35 ± 3 °C; air pressure = 4 kg/cm^2^; and aspirator pressure = −25 mbar.

### 2.5. Morphological and Physical Characterization

#### 2.5.1. Flowability

The flow ability of SEGS and SNEDDS formulations was determined by the repose angle, Carr index, and Hausner ratio in triplicate [21]. The values for the repose angle of SEGS and SNEDDS were measured using the granulate flow tester GTB equipment (Erweka, Heusenstamm, Germany). The bulk density and tap density of the SEGS and SNEDDS were determined using the tap density tester SVM equipment (Erweka, Heusenstamm, Germany) for the Carr index and Hausner ratio. The tap density was determined following 400 taps. The Carr index and Hausner ratio values were determined as follows: Carr index = [(tapped density − bulk density)/tapped density] × 100; and Hausner ratio = tapped density/bulk density [22].

#### 2.5.2. Emulsion Droplet Size

SEGS and SNEDDS formulations (200 mg) were dispersed in 20 mL distilled water by vortex mixing. The emulsion droplet size and polydispersity index (PDI) were measured using a Zetasizer Nano ZS (Malvern, UK) at a wavelength of 633 nm, a scattering angle of 90°, and 25 °C. The value for the *z*-average diameters of the emulsions was used.

#### 2.5.3. Morphological Analysis

The morphologies of the SEGS, SNEDDS, and ingredients were visualized using a scanning electron microscope (SEM) (S-4800, Hitachi, Japan). The samples were attached to a metal sample holder using adhesive tape and made electrically conductive by coating with platinum using an EMI Tech Ion Sputter (K575K) for 240 s at 15 mA.

#### 2.5.4. Solid State Characterization 

Their crystalline state properties were assessed using a powder X-ray diffraction (PXRD) spectrometer (D/MAS-2500 PC, Rigaku, Tokyo, Japan) with Cu-Kα radiation (40 mA, 40 kV) at 2-theta angles of 10–60°.

### 2.6. In Vitro Dissolution

Dissolution tests for the drug alone, SEGS, and SNEDDS containing 300 mg PLAG were performed using the USP dissolution apparatus II (Hanson, Chatsworth, CA, USA), with 900 mL pH 6.8 buffer containing 2.5% SLS at 37 ± 0.5 °C. The rotation speed of the paddle was adjusted to 100 rpm. At predetermined time intervals, an aliquot (1 mL) of the sample was collected and analysed for PLAG concentration by HPLC as described above.

### 2.7. Oral Bioavailability

Rats were orally administered with drug alone (control) and SEGS and SNEDDS formulations at a PLAG dose of 50 mg/kg. The femoral artery of the rats was cannulated using polyethylene tubing filled with heparinized isotonic saline (10 IU/mL). The SEGS and SNEDDS loaded into a small capsule (#9; Suheung, Korea) were administered orally to the rats. Following dosing of the rats, blood samples (1 mL) were collected at 0.05, 0.125, 0.25, 0.5, 1, 2, 3, 4, 6, and 8 h. The blood samples were centrifuged at 13,000× *g* for 10 min, and the plasma (300 µL) was immediately stored at −20 °C until further analysis. The concentration of PLAG in rat plasma was determined according to a previously published method [19]. Briefly, a TSQ Quantum Access MAX™ triple-stage quadrupole mass spectrometer (Thermo-Fisher, Palo Alto, CA, USA) with ESI source was used under the following conditions; Phenomenex Kinetex C-18 column (2.1 × 50 mm, 2.6 μm; Torrance, CA, USA); a mobile phase composed of solutions A (0.1% formic acid in 10 mM ammonium formate-acetonitrile (1/9, *v*/*v*)) and B (0.1% formic acid in 10 mM ammonium formate-isopropanol (1/9, *v*/*v*)) (6/4, *v*/*v*) and a flow rate of 0.3 mL/min; and detection using multiple reaction monitoring (MRM) at *m*/*z* 652.7→379.2 and *m*/*z* 655.7→382.2 for PLAG and PLAG-d3 (internal standard), respectively.

## 3. Results and Discussion

### 3.1. Component Optimization

Owing to its oily properties, PLAG is practically insoluble in water, resulting in low oral bioavailability [11]. Generally, solid formulations, containing a lipophilic drug, and liquid SMEDDS have poor flow ability [23]. The flow ability and bioavailability of oily drugs such as PLAG can be greatly improved by formulation into SEGS using fluid bed granulation. The conventional solid self-emulsifying drug delivery system was prepared with a surfactant, co-surfactant, oil, and carrier [24]. However, the SEGS were prepared without oil or co-surfactant due to the oily nature of PLAG, which easily creates a solid form.

**Figure 2:**
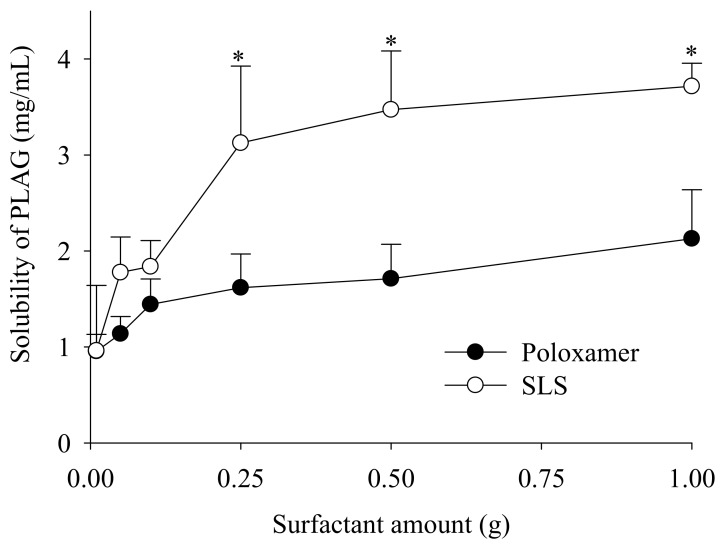
Effect of surfactant on the drug solubility. Various amounts (0.5–5%) of surfactants, such as poloxamer and sodium lauryl sulphate (SLS), were used. Each value designates the mean ± S.D. (*n* = 3). * *p* < 0.05 compared with poloxamer.

Firstly, for the selection of a solid surfactant in the present study, poloxamer (non-ionic) and SLS (anionic) were evaluated for their ability to increase the solubility of PLAG (Figure 2). SLS was selected because it showed a better solubility of PLAG as compared with poloxamer. Moreover, the solubility of PLAG increased as the amount of SLS increased; however, there was no further significant increase in drug solubility with an increase of SLS from 0.25 g to 1.0 g. SLS causes gastrointestinal adverse effects at a high oral dose [25]: thus, 0.25 g SLS was chosen due to its sufficient solubility at a lower quantity.

**Figure 3:**
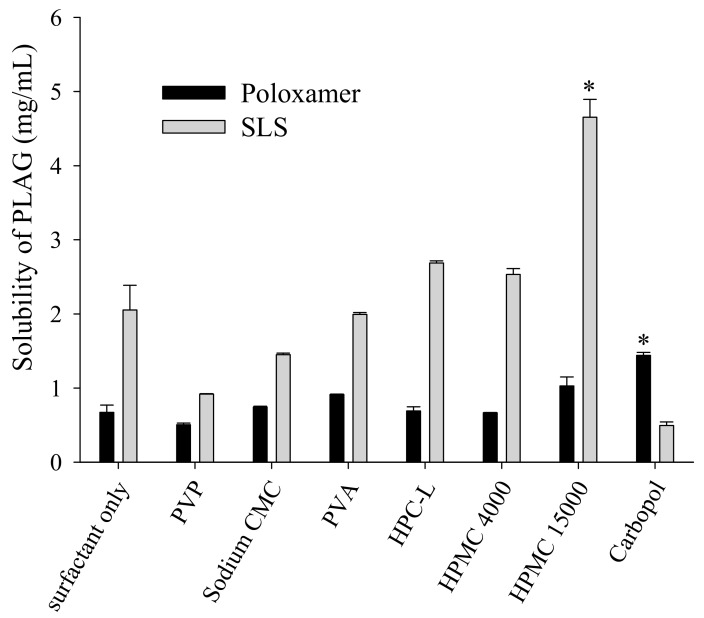
Effect of binder on the drug solubility in the surfactant solutions. An excess of PLAG was added to 10 mL distilled water containing 0.05% each binder and 1.25% surfactant solution. Each value designates the mean ± S.D. (*n* = 3). * *p* < 0.05 compared with surfactant only.

Although various methods are available for the preparation of solid self-emulsifying drug delivery systems (e.g., spray-drying, freeze-drying, rotary evaporation, and physical adsorption to solid carriers), these are unsuitable for industrial production due to the resulting low flow ability and poor blending properties [13,26]. Preparation of SEGS using fluid bed granulation is advantageous since it involves a simple granulation method and simultaneous operations (pre-blending, granulation, and drying) using a single piece of equipment [27]. The fluid bed granulation method allows the production of easily wettable small granules with good flow ability [26]; thus, the SEGS created using fluid bed granulation were expected to have good flow ability for a solid oral dosage form. Accordingly, to choose a binder for fluid bed granulation, the effect of the binder on the solubility of PLAG in 1% SLS solution was evaluated (Figure 3). The drug solubility of HPMC and cabopol were significantly higher than that of other polymers when used with SLS or poloxamer. Moreover, HPMC provided the highest drug solubility (4.65 ± 0.17 mg/mL), and thus, was chosen as the binder.

Calcium silicate and microcrystalline cellulose (MCC) were employed as the solid carriers [4,28]. MCC has been reported to provide excellent flowability and tableting properties [29,30]. In the present study, the SEGS were prepared with SLS, HPMC, calcium silicate, and MCC.

**Figure 4:**
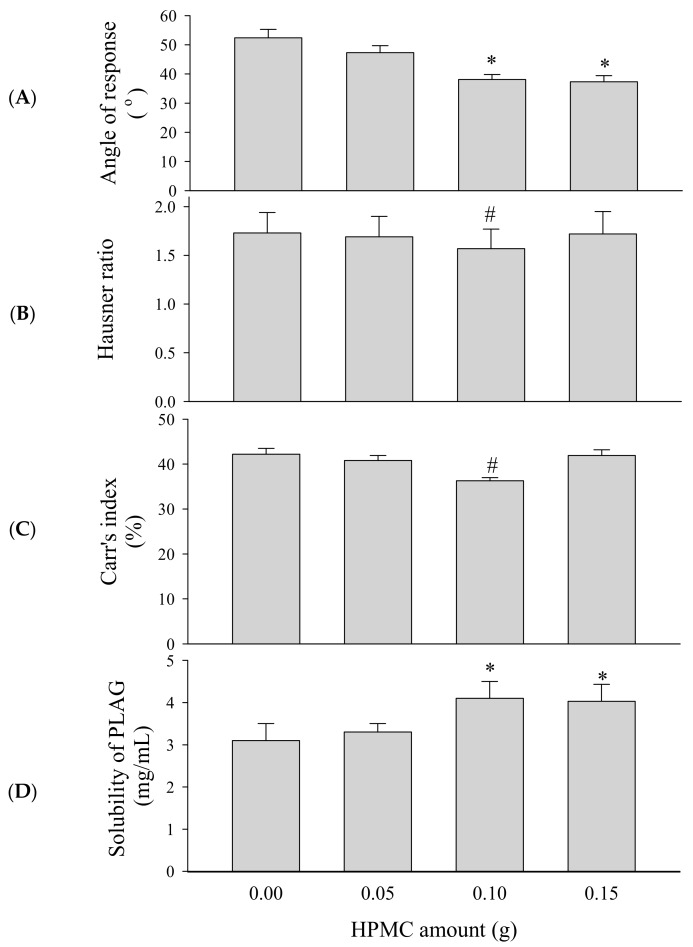
Effect of HPMC amounts on the flow ability and solubility of self-emulsifying granule system (SEGS). Each value represents the mean ± SD (*n* = 3). * *p* < 0.05 compared with HPMC amount (0 and 0.05 g); ^#^ *p* < 0.05 compared with HPMC amount (0, 0.05, and 0.15 g). (**A**) Angle of repose; (**B**) Hausner ratio; (**C**) Carr’s index; (**D**) solubility.

To select an appropriate amount of binder, SEGS were prepared with different amounts of HPMC (0–0.15 g) and fixed amounts of the drug (1.0 g), SLS (0.25 g), calcium silicate (0.5 g), and MCC (6 g) by fluid bed granulation. PLAG could be affected by fabrication process, but there was no significant change in content by fluid bed granulation. The repose angle (Figure 4A), Hausner ratio (Figure 4B), Carr index (Figure 4C), and solubility (Figure 4D) of the drug were assessed. Since flow ability is important for the preparation of oral dosage forms, SEGS were evaluated for the repose angle, Hausner ratio, and Carr index [31]. The flow ability can be known through the following parameters such as repose angle, Hausner ratio, and Carr index. Higher values of these parameters indicate relatively poor flow ability [32,33]. As the HPMC amount was increased, the repose angle decreased, but the Hausner ratio and Carr index also decreased up to 0.1 g HPMC. Moreover, the Carr index of 0.1 g HPMC was significantly lower than that of 0.15 g HPMC, indicating that 0.1 g HPMC was the appropriate amount of binder for the best flow ability of SEGS. The solubility of PLAG increased as the amount of HPMC increased. For SEGS between 0.1 g and 0.15 g HPMC, no significant difference in terms of the solubility of PLAG was detected; hence, the amount of HPMC was fixed at 0.1 g.

**Figure 5:**
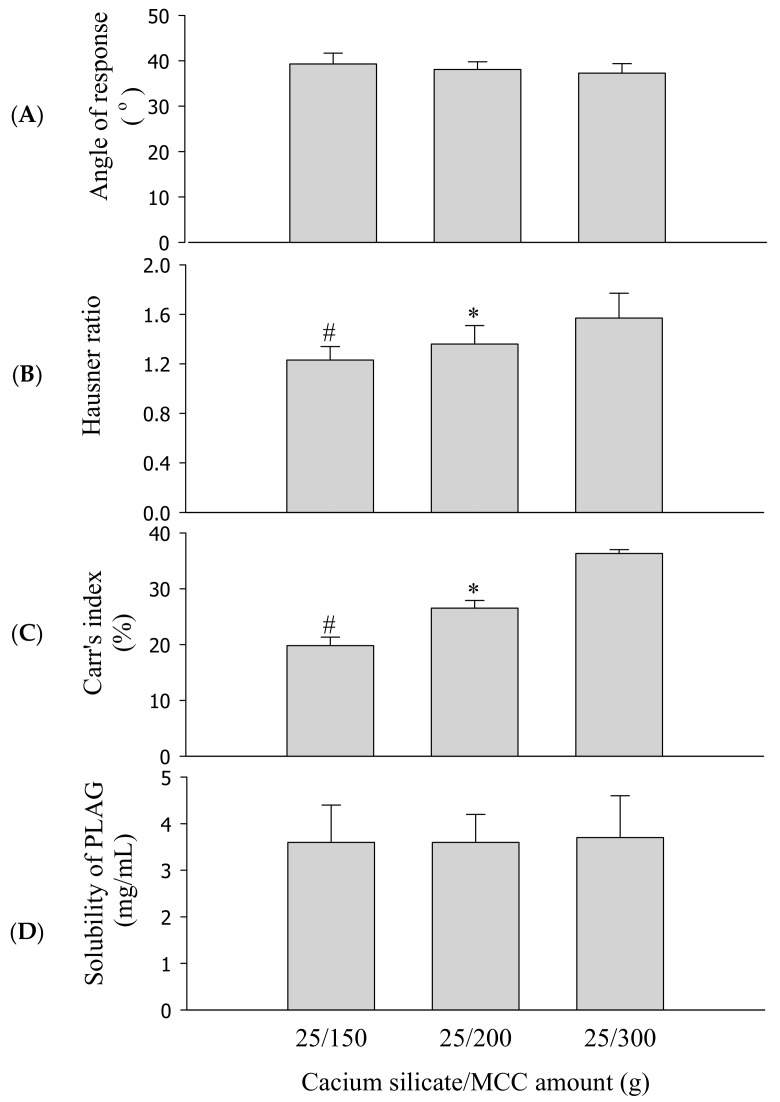
Effect of calcium silicate/microcrystalline cellulose (MCC) amounts on the flow ability and solubility of SEGS. Each value represents the mean ± SD (*n* = 3). * *p* < 0.05 compared with calcium silicate/MCC amount (25/300 g); ^#^ *p* < 0.05 compared with calcium silicate/MCC amount (25/200 and 25/300 g). (**A**) Angle of repose; (**B**) Hausner ratio; (**C**) Carr’s index; (**D**) solubility.

Solid carrier amounts of SEGS were further optimized based on the ratio of calcium silicate/MCC (0.5/3–6 g) with fixed amounts of the drug (1.0 g), SLS (0.25 g), and HPMC (0.1 g) by fluid bed granulation. Optimization of the solid carriers was performed based on their ability to produce flow ability and solubility of SEGS. In this preparation, SEGS with a ratio lower than 0.5/3 g (calcium silicate/MCC) were not prepared due to their reduced adsorption capacity of the oily drug. The repose angle (Figure 5A), Hausner ratio (Figure 5B), Carr index (Figure 5C), and solubility (Figure 5D) of the drug were assessed. As the ratio of calcium silicate/MCC was increased, SEGS were not significantly different in terms of the repose angle (Figure 5A) or solubility of the drug (Figure 5D). However, as the ratio of calcium silicate/MCC was increased, the Hausner ratio (Figure 5B) and Carr index (Figure 5C) increased. SEGS prepared at a calcium silicate/MCC ratio of 0.5/3 g provided a repose angle of approximately 39.3°, a Hausner ratio of approximately 1.23, and a Carr index of approximately 19.8%, suggesting the best flow ability. The flow ability of the SEGS is considered good when the Hausner ratio is below 1.25, the Carr index is lower than 20%, and the repose angle is between 25° and 40° [32,33]. Thus, the SEGS were considered the optimal formulation due to their excellent flow ability and solubility.

### 3.2. Comparison of SEGS and SNEDDS

SEGS were prepared with drug, SLS, HPMC, calcium silicate, and MCC at a weight ratio of 1:0.25:0.1:0.5:3 by fluid bed granulation. Moreover, the SNEDDS was prepared using the spray-drying method with the same SEGS formulation for comparison. The SEGS and SNEDDS formulations provided an emulsion droplet size of approximately 272 nm (PDI = 0.244 ± 0.013) and 277 nm (PDI = 0.250 ± 0.009), respectively. These results show excellent self-emulsifying capacity [4]. There were no significant differences (*p* > 0.05) between SEGS and SNEDDS, indicating that the solidification process in the two manufacturing methods did not lead to significant variation in the droplet size (PDI). The self-nanoemulsifying ability was well maintained in the SEGS [34].

**Figure 6:**
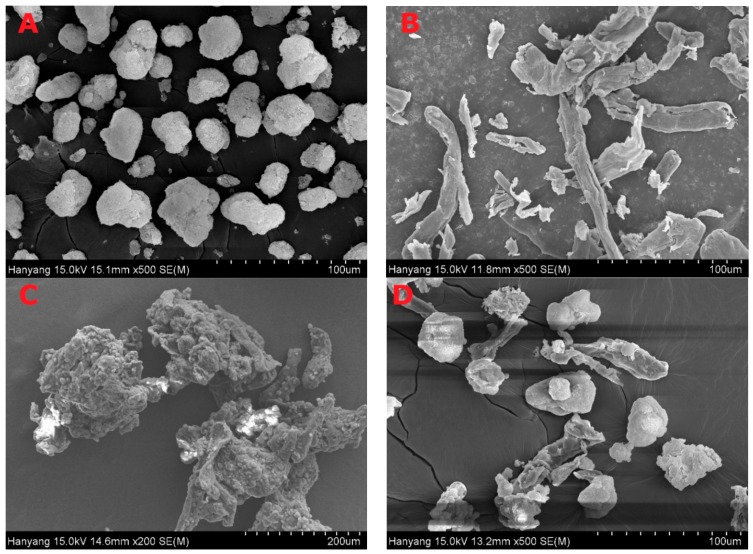
Scanning electron micrographs; (**A**) calcium silicate; (**B**) MCC; (**C**) SEGS; (**D**) solid self-nanoemulsifying drug delivery system (SNEDDS).

**Figure 7:**
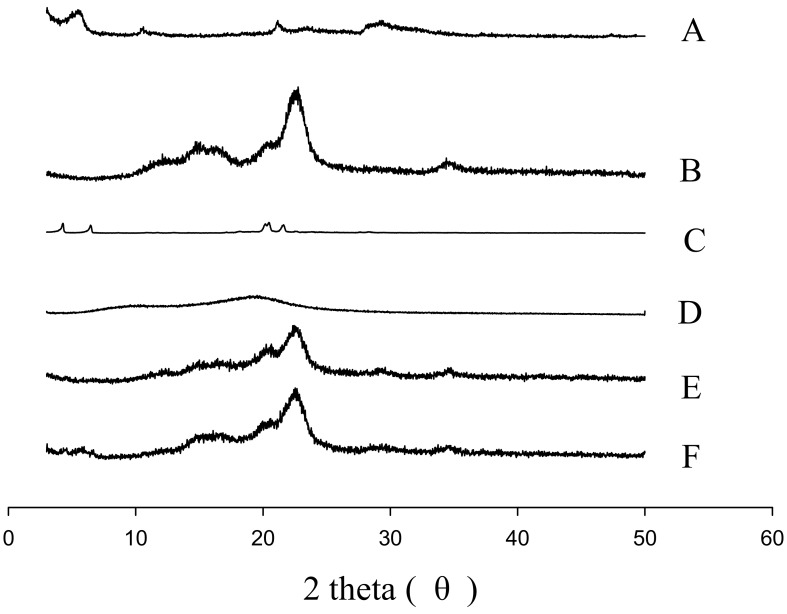
PXRD patterns: (**A**) calcium silicate; (**B**) MCC; (**C**) SLS; (**D**) HPMC; (**E**) SEGS; (**F**) SNEDDS.

Figure 6 and Figure 7 represent the SEM and PXRD patterns, respectively, of the SEGS, SNEDDS, and ingredients. Moreover, PLAG could not be determined due to its oily liquid nature. Calcium silicate (Figure 6A) and MCC (Figure 6B) had porous irregular-shaped particles and oblong particles, respectively. The SEGS (Figure 6C) and SNEDDS (Figure 6D) showed rough irregular-surfaced particles and oblong particles, respectively. With respect to the SEGS, these results suggest that the drug may exist in both the calcium silicate and MCC, but regarding the SNEDDS, the drug may exist only in the calcium silicate. Moreover, the particle size of SEGS was higher than that of SNEDDS, as shown by SEM data; thus, SEGS had better flow ability. Calcium silicate (Figure 7A), MCC (Figure 7B), and SLS (Figure 7C) showed a typical crystalline pattern [19,35,36]; however, HPMC (Figure 7D) gave no peaks by PXRD. Similarly, all typical crystalline patterns were shown for SEGS and SNEDDS (Figure 7E,F); therefore, the crystallinity of SEGS and SNEDDS was seldom influenced by the ingredients. SEGS and SNEDDS were evaluated using the Carr index, Hausner ratio, and repose angle. The Hausner ratios and Carr indices of SEGS and SNEDDS were 1.23 vs. 2.07 and 19.8% vs. 43.5%, respectively. Moreover, SNEDDS showed a repose angle of 49.2 ± 2.9°, indicating poor flow ability. The SEGS showed a repose angle (38.1 ± 1.8°) less than 40, indicating good flow ability. The results show better flow ability of SEGS by fluid bed granulation than SNEDDS by the spray-drying method [37].

**Figure 8:**
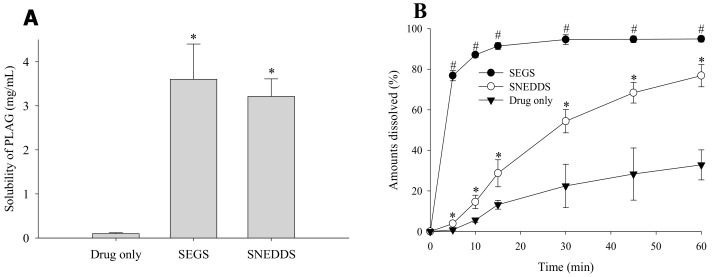
Drug solubility (**A**) and dissolution profile (**B**) of SEGS and SNEDDS in the pH 6.8 buffer containing 2.5% SLS. The SEGS and SNEDDS were composed of PLAG/SLS/HPMC/calcium silicate/MCC at the weight ratio of 1:0.25:0.1:0.5:3. Each value designates the mean ± S.D. (*n* = 6). * *p* < 0.05 compared with drug only; ^#^ *p* < 0.05 compared with drug only and SNEDDS.

The effect of SEGS and SNEDDS on the solubility and dissolution of the drug was investigated (Figure 8). The solubility of PLAG alone, SEGS, and SNEDDS were approximately 0, 3.6 ± 0.8, and 3.2 ± 0.4 mg/mL, respectively (Figure 8A). SEGS and SNEDDS enhanced the solubility of the drug by approximately 36- and 32-fold, as compared with the drug alone. There were no significant differences between SEGS and SNEDDS. Moreover, their dissolutions were evaluated in pH 6.8 buffer containing 2.5% SLS (Figure 8B). The SEGS and SNEDDS enhanced the dissolution of PLAG in both the dissolution mediums. The SEGS gave a significantly more rapid and higher dissolution rate of PLAG than SNEDDS and drug alone in pH 6.8 (91.4 ± 1.7 vs. 28.7 ± 6.7 vs. 13.2 ± 2.2% at 15 min) buffer. Moreover, the SNEDDS gave a significantly higher dissolution rate of PLAG than the drug only in both dissolution mediums. The SEGS exhibited a rapid release and maximum dissolution of PLAG within 15 min, while SNEDDS took 45 min to show the maximum dissolution. In the case of SNEDDS, the MCC was post-mixed with SNEDDS, causing low exposure of the drug to MCC in the final powder. However, in the case of SEGS, the drug/surfactant/binder in solution was coated on the calcium silicate/MCC, causing a high drug to MCC ratio, which led to a better exposure in dissolution media for faster wettability and release of PLAG. Thus, the wettability of SEGS using fluid bed granulation was better than that of SNEDDS using the spray-drying method [26].

**Figure 9:**
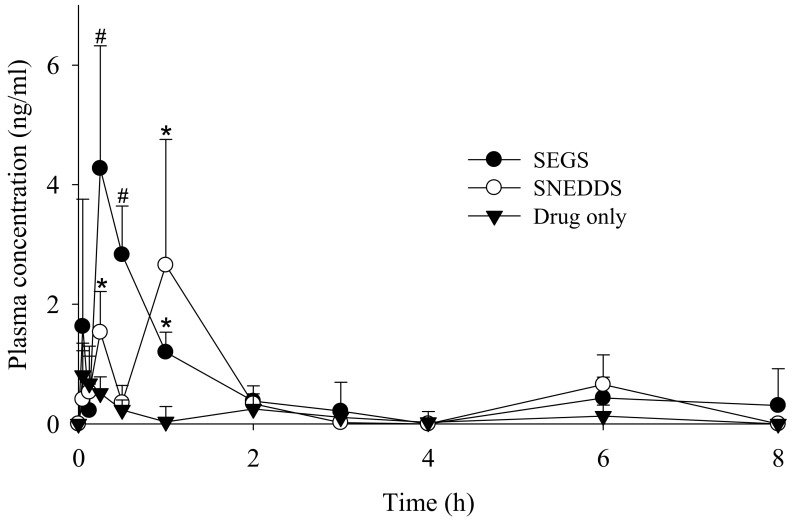
Mean plasma level-time profiles of PLAG after oral administration of drug alone, SEGS and SNEDDS at a dose equivalent to 50 mg/kg PLAG in rats. Each value designates the mean ± S.D. (*n* = 6). The SEGS and SNEDDS were composed of PLAG/SLS/HPMC/calcium silicate/MCC at the weight ratio of 1:0.25:0.1:0.5:3. * *p* < 0.05 compared with drug only; ^#^ *p* < 0.05 compared with drug only and SNEDDS.

Figure 9 shows the change in mean plasma concentration of PLAG following oral administration of PLAG alone, SEGS, and SNEDDS at a dose of 50 mg/kg PLAG in rats. The SEGS and SNEDDS showed a significantly higher plasma concentration as compared with PLAG alone. It was observed that the SEGS and SNEDDS produced double peak profiles, unlike the single peak of PLAG alone. This phenomenon may be explained by the presence of two or more absorption sites in the intestines and hepatoenteric circulation of the increased bioavailable PLAG in the SEGS and SNEDDS [38]. Moreover, from 15 to 30 min, the plasma concentration in the SEGS was significantly higher than that in the SNEDDS. Further, at 1 h, the plasma concentration in the SNEDDS was significantly higher than that in the SEGS. The plasma level-time profiles of PLAG showed that SEGS were more rapidly absorbed as compared with SNEDDS, which is similar to our dissolution findings. The corresponding pharmacokinetic parameters are given in Table 1. In comparison with each formulation, the SEGS and SNEDDS had significantly a higher area under the curve (AUC) and maximum serum concentration (C_max_) as compared with PLAG alone. Moreover, the C_max_ value for the SEGS was higher than that of the SNEDDS; however, the difference was not significant. The SEGS had a faster time to maximum concentration (T_max_) as compared with the SNEDDS; however, there were no significant differences in T_max_ value among all formulations. The enhanced oral bioavailability of the SEGS and SNEDDS results from an increased solubility and dissolution [39,40]. Moreover, SEGS produced by fluid bed granulation gave a higher and faster oral bioavailability of the oily drug than that of the SNEDDS produced by spray-drying. This may be due to the rapid dissolution rate and increased dissolution of PLAG using fluid bed granulation. Our results suggest that the bioavailability enhancement by SEGS was highly dependent upon the solubility and dissolution correlated with their manufacturing method.

## 4. Conclusions

From the comparison of SEGS and SNEDDS, the SEGS, which was prepared using fluid bed granulation, improved the flow ability, solubility, dissolution, and oral bioavailability of PLAG, in compared to the SNEDDS prepared using the spray-drying method. Thus, SEGS can be recommended as a potential oral solid dosage form for PLAG.

## Figures and Tables

**Table 1:** Pharmacokinetic parameters after oral administration of drug alone, SEGS and SNEDDS at a dose equivalent to 50 mg/kg drug in rats.

Parameter	Drug	SEGS	SNEDDS
T_max_ (h)	0.50 ± 0.65	0.26 ± 0.18	1.00 ± 0.77
C_max_ (ng/mL)	0.81 ± 0.42	4.27 ± 3.72 *	2.66 ± 1.60 *
AUC (h·ng/mL)	0.98 ± 0.83	4.65 ± 1.05 *	4.76 ± 2.51 *

Each value represents the mean ± S.D. (*n* = 8). SEGS and SNEDDS were composed of PLAG/SLS/HPMC/calcium silicate/MCC at the weight ratio of 1:0.25:0.1:0.5:3. * *p* < 0.05 compared with drug.
